# Gauze Packing Objectionable

**Published:** 1902-12

**Authors:** 


### Gauze Packing Objectionable.

The use of iodoform gauze for packing the pelvis or limited parts of the abdomen has become so common that it will be hard to abandon it—whatever its disadvantages. That it should be discarded, especially in operations for appendicitis, is maintained strenuously by Dr. R. T. Morris, of New York. He claims that if one would take ten strong, healthy policemen from the street at nine o'clock some evening and put half a yard of gauze into each of their abdominal cavities, under even the most careful antiseptic precautions, by nine o'clock the next evening he would find such a lethal condition as to be compelled to use the oft-heard expression: "I did not get them in time." He maintains that the use of gauze was introduced as a rude method for obtaining the desired result, but is in truth open to the gravest objections. By adding iodoform to the gauze he believes we add another element of distress, for the patient is thereby exposed to insidious iodoform poisoning. "In iodoform poisoning," he remarks, "the wound looks uncommonly well, but the patient does not." He dilates eloquently upon the distress which is inexcitably associated with the presence of a mass of gauze (which is like any other foreign body) in the delicate peritoneal cavity, to say nothing of the misery incidental to its removal and replacement. He accuses the packing of causing intestinal obstruction by direct pressure, of setting up adhesions which are sure to be a source of annoyance and even danger in after life, and, indirectly, of leaving a permanently weak spot in the patient's abdominal wall with consequent ventral hernia. Yet, in spite of his formidable indictment, Dr. Morris does not feel justified in urging the instant abandonment of gauze packing, which forms too integral a part of present-day surgical practice, but he strongly advises working ever toward a point when it may be given up; which will be as soon as experience proves that this step can be taken with safety. He invites every operator to study the statistics of surgeons who employ gauze and those who employ it not, in order to arrive at a trustworthy conclusion as to its efficacy for the purpose for which it is used.—*Am. Jour. Surg. and Gynecology.*

